# Supplementary material for: Clonal evolution in myelodysplastic syndromes
Source: Nat Commun. 2017 Apr 21;8:15099. doi: 10.1038/ncomms15099 (PMC5530598; doi:10.1038/ncomms15099)
Supplement: Supplementary Information — Supplementary Figures and Supplementary Tables [file ncomms15099-s1.pdf]

Supplementary Table 1. Trusight myeloid gene panel

54 driver genes				
ABL1	CEBPA	HRAS	MYD88	SF3B1
ASXL1	CSF3R	IDH1	NOTCH1	SMC1A
ATRX	CUX1	IDH2	NPM1	SMC3
BCOR	DNMT3A	IKZF1	NRAS	SRSF2
BCORL1	ETV6/TEL	JAK2	PDGFRA	STAG2
BRAF	EZH2	JAK3	PHF6	TET2
CALR	FBXW7	KDM6A	PTEN	TP53
CBL	FLT3	KIT	PTPN11	U2AF1
CBLB	GATA1	KRAS	RAD21	WT1
CBLC	GATA2	MLL	RUNX1	ZRSR2
CDKN2A	GNAS	MPL	SETBP1	

Supplementary Table 2. Gene panel targeted-capture sequencing

72 driver genes				
ASXL1	CUX1	IDH2	PHF6	SMC1A
ASXL2	DCLRE1C	IRF1	PIGA	SMC3
ATM	DDX41	JAK2	PIGT	SRSF2
ATRX	DNMT3A	KDM6A	PPM1D	STAG1
BCOR	EP300	KIT	PRPF8	STAG2
BCORL1	ETNK1	KRAS	PTPN11	STAT3
BRCC3	ETV6	LUC7L2	RAD21	TET2
CALR	EZH2	MLL2	RIT1	TP53
CBL	FANCA	MPL	RUNX1	U2AF1
CDKN2A	FANCM	MRE11A	SETBP1	U2AF2
CEBPA	FLT3	NF1	SETD2	WT1
CHEK2	GATA2	NFE2	SF1	ZRSR2
CREBBP	GNAS	NPM1	SF3A1	
CSF3R	GNB1	NRAS	SF3B1	
CTCF	IDH1	PDS5B	SH2B3	

Supplementary Table 3. Results SNP array

Patient	Months from BL	Aberration (% of cells)	Aberration according to ISCN (hg19 reference)
**UPN01**	2	del(5q) (70%)	5q15q33.2(95.804.838-154.408.872)x1~2
	119	del(5q) (90%)	5q15q33.2(95.804.838-154.408.872)x1~2
**UPN02**	BL	CN-LOH4q (75%)	4q21.23qter(85.662.390-190.921.709)x2 hmz
	60	CN-LOH4q (90%)	4q21.23qter(85.662.390-190.921.709)x2 hmz
**UPN03**	BL	no abn	(1-22)x2,(XY)x1
	93	no abn	(1-22)x2,(XY)x1
**UPN04**	BL	no abn	(1-22)x2,(XY)x1
	72	no abn	(1-22)x2,(XY)x1
**UPN05**	BL	trisomy 8 (20%)	(8)x2~3
		loss *ETV6* (90%)	12p13.2(11867287-12027012)x1
	23	loss *ETV6* (40%)	12p13.2(11867287-12027012)x1
	48	no abn	(1-22)x2,(XY)x1
	79	loss *ETV6* (90%)	12p13.2(11867287-12027012)x1
	84	loss *ETV6* (90%)	12p13.2(11867287-12027012)x1
		gain *KMT2A* (60%)	11q23.3(118338477-118354345)x3
**UPN06**	BL	trisomy 21 (50%)	(21)x2~3
	20	trisomy 21 (40%)	(21)x2~3
	57	trisomy 8 (60%)	(8)x3
		CN-LOH14q (100%)	14q11.2qter(23431738-107285437)x2 hmz
		trisomy 21 (75%)	(21)x3
	63	trisomy 8 (90%)	(8)x3
		CN-LOH14q (95%)	14q11.2qter(23431738-107285437)x2 hmz
		trisomy 21 (95%)	(21)x3
	64	trisomy 8 (100%)	(8)x3
		CN-LOH14q (95%)	14q11.2qter(23431738-107285437)x2 hmz
		trisomy 21(100%)	(21)x3
**UPN07**	BL	no abn	(1-22)x2,(XY)x1
	23	no abn	(1-22)x2,(XY)x1
	38	trisomy 8 (40%)	(8)x2~3
	44	complex including trisomy 8 (20-30%)	(3,5,7,9,10,12,16,17,22)x1~2
			4q13.3q24(72018147-102866413)x2~3
			4q24qter(102866413-191000000)x1~2
			(8)x2~3
**UPN08**	BL	del(5q) (90%)	5q21.3q34(82169433-161991376)x1
	112	no abn	(1-22,X)x2
**UPN09**	BL	del(5q) (60%)	5q14.3q34(86890558-162823229)x1~2
	53	no abn	(1-22,X)x2
**UPN10**	3	del(5q) (10%)	5q15q33.3(98131505-156829473)x1~2
		del(13q) (10%)	13q13.1q14.3(32377128-53622642)x1~2
	49	no abn	(1-22,X)x2
**UPN11**	BL	gain *ERG* 21q (90%)	21q22.13(39593444-39950442)x3
	29	gain *ERG* 21q (100%)	21q22.13(39593444-39950442)x3

UPN indicates unique patient number; hmz, homozygous; CN-LOH, copy-neutral loss of heterozygosity; no abn, no abnormalities; BL, baseline

Supplementary Table 4: Detected variants in T-cells

Patient	no.	Gene	Type of variant	Chromosome	Start co-ordinate of variant (GRCh37/hg19)	End co-ordinate of variant (GRCh37/hg19)	Reference sequence	Observed sequence	Transcript ID	Coding sequence change	Amino acid change	VAF (%)
**UPN03**	1	ZNF493	nonsynonymous SNV	chr19	21607599	21607599	G	A	NM_175910	c.G1754A	p.C585Y	7.4
	2	ZNF493	nonsynonymous SNV	chr19	21606245	21606245	C	G	NM_175910	c.C400G	p.Q134E	10.8
	3	ZNF716	nonsynonymous SNV	chr7	57529057	57529057	C	A	NM_001159279	c.C890A	p.T297K	8.7
	4	ZNF721	nonsynonymous SNV	chr4	435743	435743	G	T	NM_133474	c.C2513A	p.T838K	7.3
	5	ZNF732	nonsynonymous SNV	chr4	266059	266059	G	T	NM_001137608	c.C584A	p.T195K	10.0
**UPN04**	-	-	-	-	-	-	-	-	-	-	-	-
**UPN05**	-	-	-	-	-	-	-	-	-	-	-	-
**UPN07**	1	ABCF1	nonsynonymous SNV	chr6	30539290	30539290	C	T	NM_001025091	c.C26T	p.P9L	17.0
	2	ERC2	nonsynonymous SNV	chr3	55733486	55733486	A	G	NM_015576	c.T2761C	p.Y921H	10.2
	3	MYLK	nonsynonymous SNV	chr3	123419020	123419020	C	T	NM_053026	c.G3088A	p.A1030T	7.5
	4	OR10G7	nonsynonymous SNV	chr11	123909011	123909011	C	T	NM_001004463	c.G698A	p.R233K	7.1
	5	OR10G8	nonsynonymous SNV	chr11	123900495	123900495	A	C	NM_001004464	c.A166C	p.T56P	11.4
	6	SERPINA3	nonsynonymous SNV	chr14	95085756	95085756	A	C	NM_001085	c.A868C	p.M290L	10.0
	7	SERPINA3	nonsynonymous SNV	chr14	95085757	95085757	T	A	NM_001085	c.T869A	p.M290K	9.4
	8	TMEM67	frameshift insertion	chr8	94772148	94772148	-	T	NM_001142301	c.91dupT	p.G30fs	7.1
	9	ZNF107	nonsynonymous SNV	chr7	64167829	64167829	T	C	NM_001282360	c.T1258C	p.S420P	7.6
	10	ZNF138	nonsynonymous SNV	chr7	64292467	64292467	C	G	NM_001271649	c.C406G	p.Q136E	8.3
	11	ZNF208	nonsynonymous SNV	chr19	22154859	22154859	C	T	NM_007153	c.G2977A	p.V993I	7.3
	12	ZNF267	nonsynonymous SNV	chr16	31927543	31927543	G	A	NM_003414	c.G1973A	p.R658K	9.8
	13	ZNF431	nonsynonymous SNV	chr19	21365885	21365885	T	A	NM_133473	c.T779A	p.F260Y	7.0
	14	ZNF678	nonsynonymous SNV	chr1	227842419	227842419	C	A	NM_178549	c.C633A	p.D211E	8.0
	15	ZNF695	nonsynonymous SNV	chr1	247151018	247151018	T	G	NM_020394	c.A799C	p.T267P	11.2
	16	ZNF714	nonsynonymous SNV	chr19	21300069	21300069	T	A	NM_182515	c.T599A	p.F200Y	7.6
	17	ZNF721	nonsynonymous SNV	chr4	436658	436658	A	T	NM_133474	c.T1598A	p.V533E	7.3
	18	ZNF721	nonsynonymous SNV	chr4	435773	435773	C	T	NM_133474	c.G2483A	p.R828K	7.0
	19	ZNF721	nonsynonymous SNV	chr4	436583	436583	G	T	NM_133474	c.C1673A	p.T558K	7.1
	20	ZNF721	nonsynonymous SNV	chr4	437330	437330	A	T	NM_133474	c.T926A	p.V309E	7.1
	21	ZNF732	nonsynonymous SNV	chr4	266078	266078	C	T	NM_001137608	c.G565A	p.A189T	9.7
	22	ZNF732	nonsynonymous SNV	chr4	266059	266059	G	T	NM_001137608	c.C584A	p.T195K	9.8
	23	ZNF91	nonsynonymous SNV	chr19	23542561	23542561	T	C	NM_001300951	c.A3124G	p.R1042G	7.3
	24	ZNF99	nonsynonymous SNV	chr19	22940859	22940859	T	C	NM_001080409	c.A1852G	p.K618E	11.2
**UPN08**	1	ZNF716	nonsynonymous SNV	chr7	57529057	57529057	C	A	NM_001159279	c.C890A	p.T297K	7.6

UPN indicates unique patient number; Chr., chromosome; SNV, single nucleotide variant.

Supplementary Table 5. Primer strategy

PCR	Direction	Primer build up	Sequence primer (5'--> 3')
PCR1	Forward	CS1 - target specific sequence	ACACTGACGACATGGTTCTACAxxx
	Reverse	CS2 - target specific sequence	TACGGTAGCAGAGACTTGGTCTxxx
PCR2_A	Forward	A adapter - barcode - CS1	CCATCTCATCCCTGCGTGTCTCCGACTCAGyyyACACTGACGACATGGTTCTACA
	Reverse	trP1 adapter - CS2	CCTCTCTATGGGCAGTCGGTGATTACGGTAGCAGAGACTTGGTCT
PCR2_B	Forward	trP1 adapter - CS1	CCTCTCTATGGGCAGTCGGTGATACACTGACGACATGGTTCTACA
	Reverse	A adapter - barcode - CS2	CCATCTCATCCCTGCGTGTCTCCGACTCAGyyyTACGGTAGCAGAGACTTGGTCT

CS: common sequence
xxx: Target specific primer, see Supplementary data 4
yyy: Barcode primer, see Supplementary Table 6

Supplementary Table 6. Barcode primer sequences

Barcode no.	Barcode Sequence	Barcode no.	Barcode Sequence
Ion Xpress_1	CTAAGGTAAC	Ion Xpress_26	TTACAACCTC
Ion Xpress_2	TAAGGAGAAC	Ion Xpress_27	AACCATCCGC
Ion Xpress_3	AAGAGGATTC	Ion Xpress_28	ATCCGGAATC
Ion Xpress_4	TACCAAGATC	Ion Xpress_29	TCGACCACTC
Ion Xpress_5	CAGAAGGAAC	Ion Xpress_30	CGAGGTTATC
Ion Xpress_6	CTGCAAGTTC	Ion Xpress_31	TCCAAGCTGC
Ion Xpress_7	TTCGTGATTC	Ion Xpress_32	TCTTACACAC
Ion Xpress_8	TTCCGATAAC	Ion Xpress_33	TTCTCATTGAAC
Ion Xpress_9	TGAGCGGAAC	Ion Xpress_34	TCGCATCGTTC
Ion Xpress_10	CTGACCGAAC	Ion Xpress_35	TAAGCCATTGTC
Ion Xpress_11	TCCTCGAATC	Ion Xpress_36	AAGGAATCGTC
Ion Xpress_12	TAGGTGGTTC	Ion Xpress_37	CTTGAGAATGTC
Ion Xpress_13	TCTAACGGAC	Ion Xpress_38	TGGAGGACGGAC
Ion Xpress_14	TTGGAGTGTC	Ion Xpress_39	TAACAATCGGC
Ion Xpress_15	TCTAGAGGTC	Ion Xpress_40	CTGACATAATC
Ion Xpress_16	TCTGGATGAC	Ion Xpress_41	TTCCACTTCGC
Ion Xpress_17	TCTATTCGTC	Ion Xpress_42	AGCACGAATC
Ion Xpress_18	AGGCAATTGC	Ion Xpress_43	CTTGACACCGC
Ion Xpress_19	TTAGTCGGAC	Ion Xpress_44	TTGGAGGCCAGC
Ion Xpress_20	CAGATCCATC	Ion Xpress_45	TGGAGCTTCCTC
Ion Xpress_21	TCGCAATTAC	Ion Xpress_46	TCAGTCCGAAC
Ion Xpress_22	TTCGAGACGC	Ion Xpress_47	TAAGGCAACCAC
Ion Xpress_23	TGCCACGAAC	Ion Xpress_48	TTCTAAGAGAC
Ion Xpress_24	AACCTCATTC	Ion Xpress_49	TCCTAACATAAC
Ion Xpress_25	CCTGAGATAC	Ion Xpress_50	CGGACAATGGC

No. indicates number

Supplementary Table 7. PCR1 protocols

PCR1 program 1			
**Step**	**Temperature**	**Time**	**Cycles**
1	98˚C	30 sec	1
2	98˚C	10 sec	10 cycles touchdown + 20 cycles at 58˚C
	63˚C → 58˚C (delta -0.5˚C)	15 sec	
	72˚C	10 sec	
3	72˚C	2.00 min	1
	4˚C	hold	
**PCR1 program 2**			
**Step**	**Temperature**	**Time**	**Cycles**
1	98˚C	30 sec	1
2	98˚C	10 sec	15 cycles touchdown + 15 cycles at 58˚C
	73˚C → 58˚C (delta -1˚C)	15 sec	
	72˚C	10 sec	
3	72˚C	2.00 min	1
	4˚C	hold	
**PCR1 program 3**			
**Step**	**Temperature**	**Time**	**Cycles**
1	98˚C	30 sec	1
2	98˚C	10 sec	10 cycles touchdown + 20 cycles op 63˚C
	73˚C → 63˚C (delta -1˚C)	15 sec	
	72˚C	10 sec	
3	72˚C	2.00 min	1
	4˚C	hold	

Supplementary Table 8. PCR2 protocol

PCR2 program			
**Step**	**Temperature**	**Time**	**Cycles**
1	98˚C	30 sec	1
2	98˚C	10 sec	10 cycles
	60˚C	15 sec	
	72˚C	10 sec	
3	72˚C	2.00 min	1
	4˚C	hold	

Supplementary Table 9. Analysis of 8 mutations in 10 healthy donors

Mutation	Donor 1	Donor 2	Donor 3	Donor 4	Donor 5	Donor 6	Donor 7	Donor 8	Donor 9	Donor 10
CEP89 p.P48R (VAF %)	0.04	0.08	0.07	0.02	0.10	0.08	0.11	0.18	0.11	0.06
DOCK5 p.M800R (VAF %)	0.00	0.00	0.01	0.00	0.00	0.00	0.00	0.00	0.00	0.00
OR14I1 p.R120L (VAF %)	0.01	0.00	0.00	0.00	0.00	0.01	0.02	0.01	0.00	0.00
PALLD p.P51S (VAF %)	0.01	0.02	0.02	0.02	0.00	0.00	0.02	0.02	0.01	0.02
RAD50 p.D69Y (VAF %)	0.00	0.00	0.01	0.00	0.00	0.00	0.00	0.00	0.00	0.01
RASA1 p.A170P (VAF %)	0.01	0.01	0.01	0.01	0.01	0.01	0.02	0.01	0.01	0.01
RELN p.R3397W (VAF %)	0.02	0.01	0.04	0.02	0.03	0.01	0.03	0.01	0.02	0.02
TP53 p.R141L (VAF %)	0.01	0.00	0.01	0.00	0.00	0.00	0.00	0.01	0.00	0.00

100% specificity with a cut-off of 0.20%

Supplementary Table 10. Dilution series of three different SNPs

	Percentage DNA with SNP												
**SNPs**	**0**	**0.05**	**0.1**	**0.2**	**0.4**	**0.8**	**1.6**	**3.1**	**6.3**	**12.5**	**25**	**50**	**100**
CLDN18 p.M1V heterozygous (VAF %)	0.01	0.05	0.05	0.1	0.14	0.3	0.6	1	2	5	10	22	49
TET2 p.N1774S heterozygous (VAF %)	-	0.02	0.05	0.07	0.14	0.17	0.51	0.99	2	4	9	18	41
TET2 p.I1762V homozygous (VAF %)	0	0.04	0.07	0.19	0.29	0.44	1.04	2	4	9	19	41	100

- indicates SNP does not exceed noise: the SNP is not the second highest base at this position

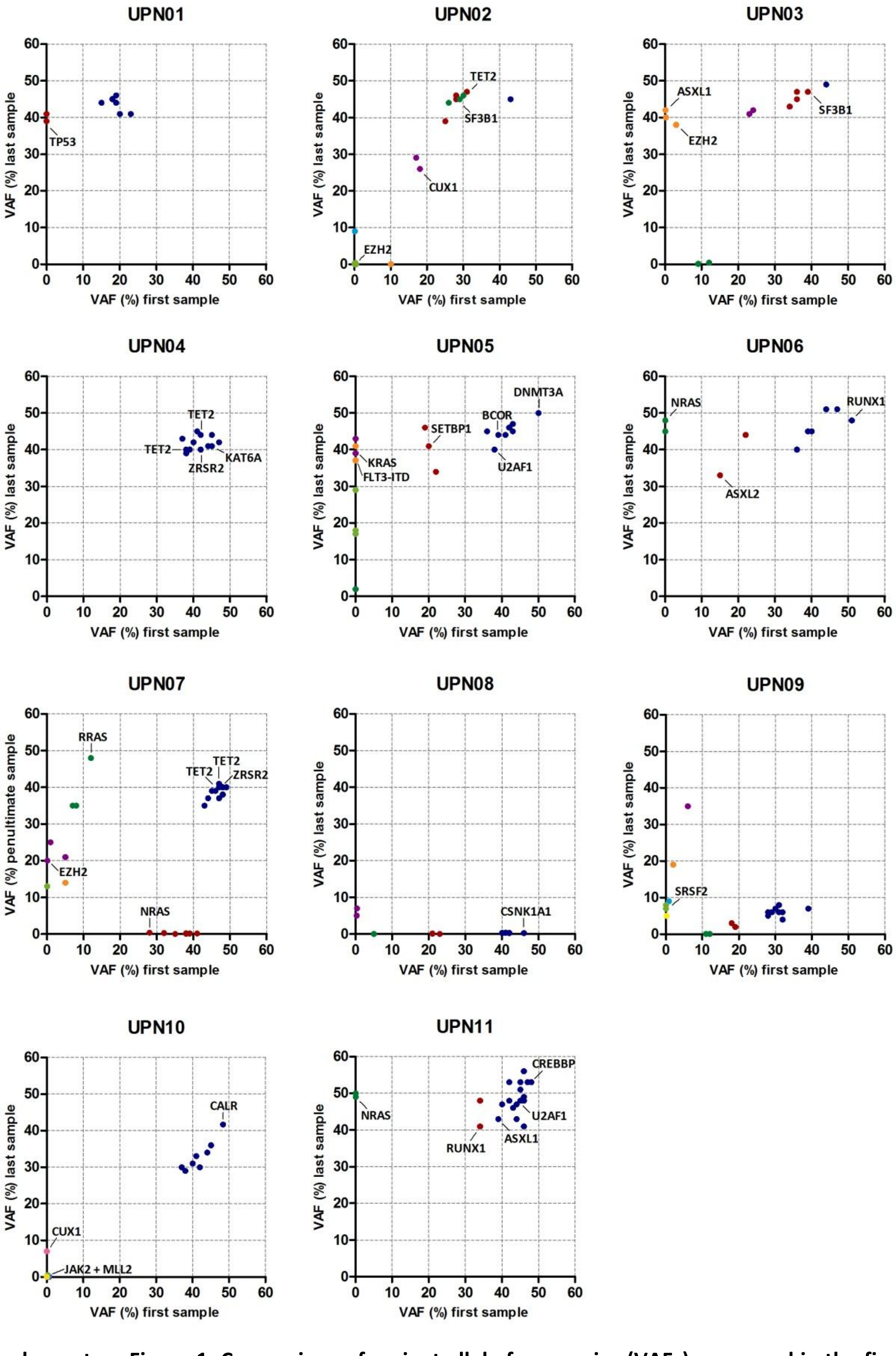

Supplementary Figure 1. Comparison of variant allele frequencies (VAFs) measured in the first and last bone marrow sample of each patient. Variants located on the X- or Y-axis indicate mutations only present in the first or last sample. Many clones can already be distinguished by comparing these two samples. However, clonal composition cannot be fully resolved, as some mutations belonging to different (sub)clones cluster together (UPN02, UPN05, UPN09), and some mutations will be missed (UPN02, UPN10) when only the first and last sample are investigated, without intermediate time points. VAFs of mutations in regions harboring a copy number variation are corrected for local ploidy. For UPN07, the penultimate sample was used, since the last sample had a very complex karyotype. Mutations within the same clone were colored identical.

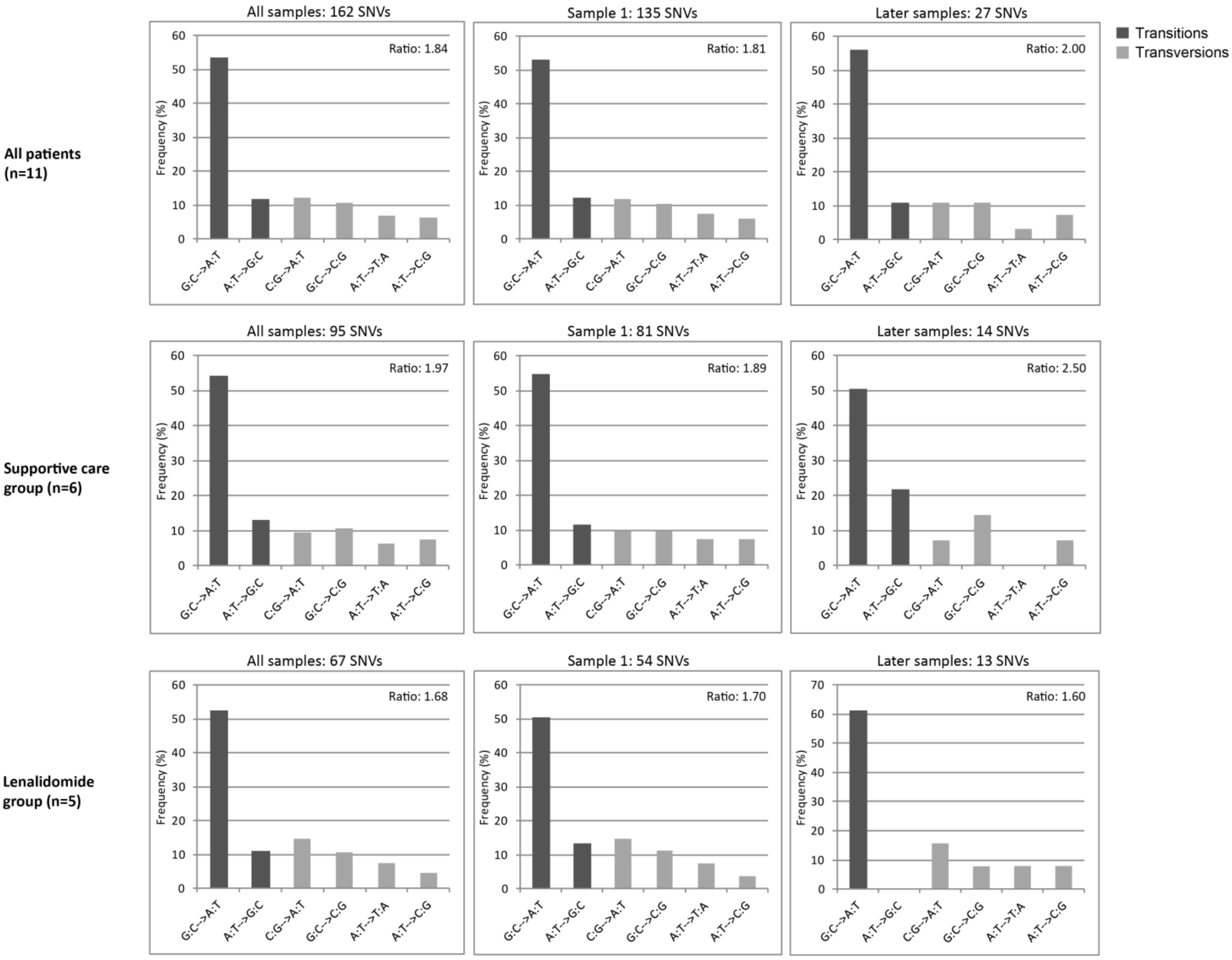

Supplementary Figure 2. Comparison of the type of mutations in early vs. later time points. No major differences were observed when comparing the type of mutations present in the first sample (variant allele frequency>0.2%) compared to mutations that arise at later time points. Also, when comparing the two treatment groups no major differences were observed. The transition/transversion ratio is indicated in each graph.

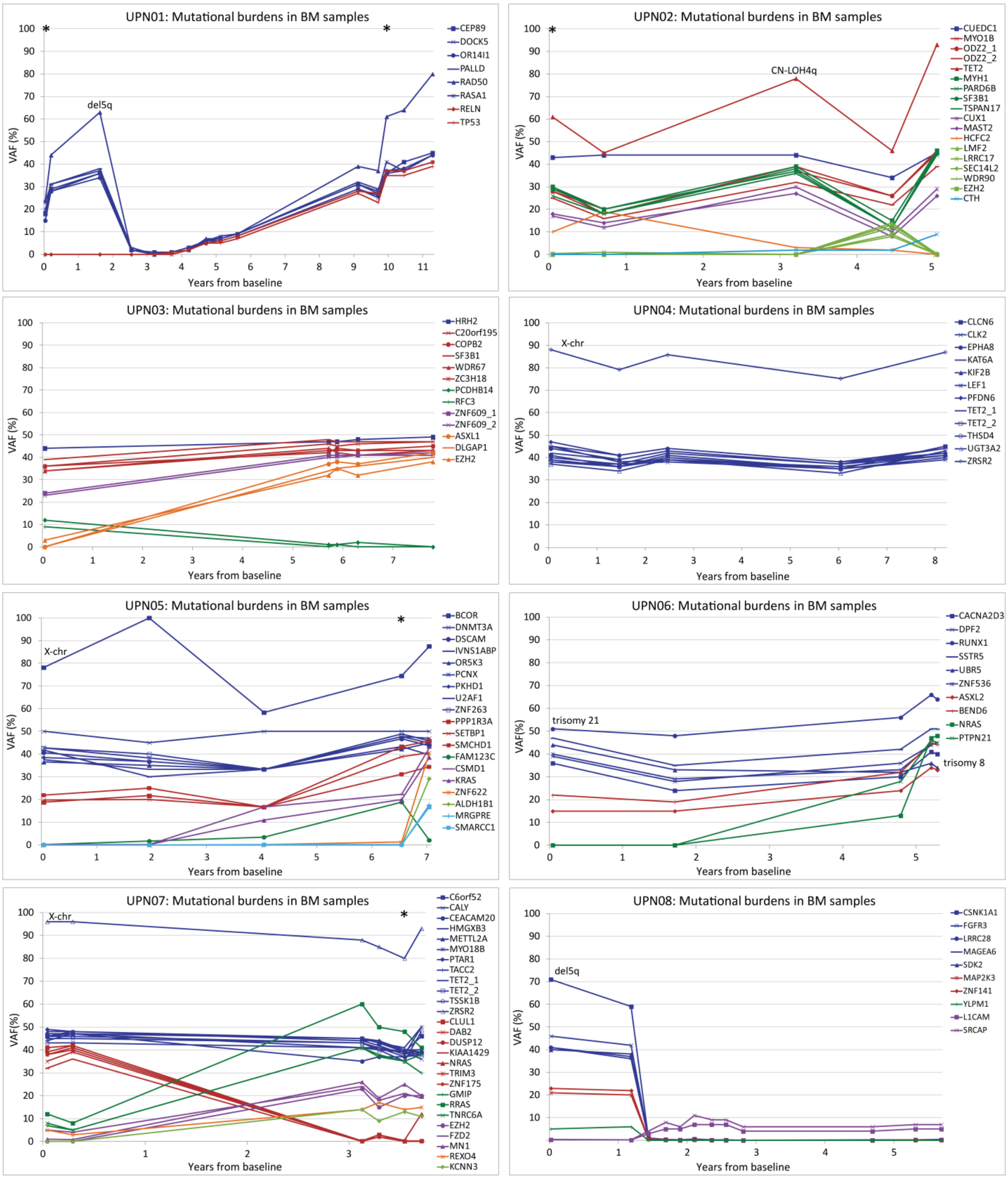

Supplementary Figure 3. Mutational burdens in bone marrow (BM) samples. Graphs showing the variant allele frequencies (VAFs) measured using amplicon-based targeted deep sequencing (not corrected for local ploidy). For patient UPN01-UPN08, the patterns of all mutations are plotted in time and mutations are color-coded to match the (sub)clones as depicted in figures 2 and 3. Indicated is also whether a variant locates to a region affected by a copy number alteration or is located to the X-chromosome in case of a male patient. *indicates amplified DNA sample used for targeted deep sequencing

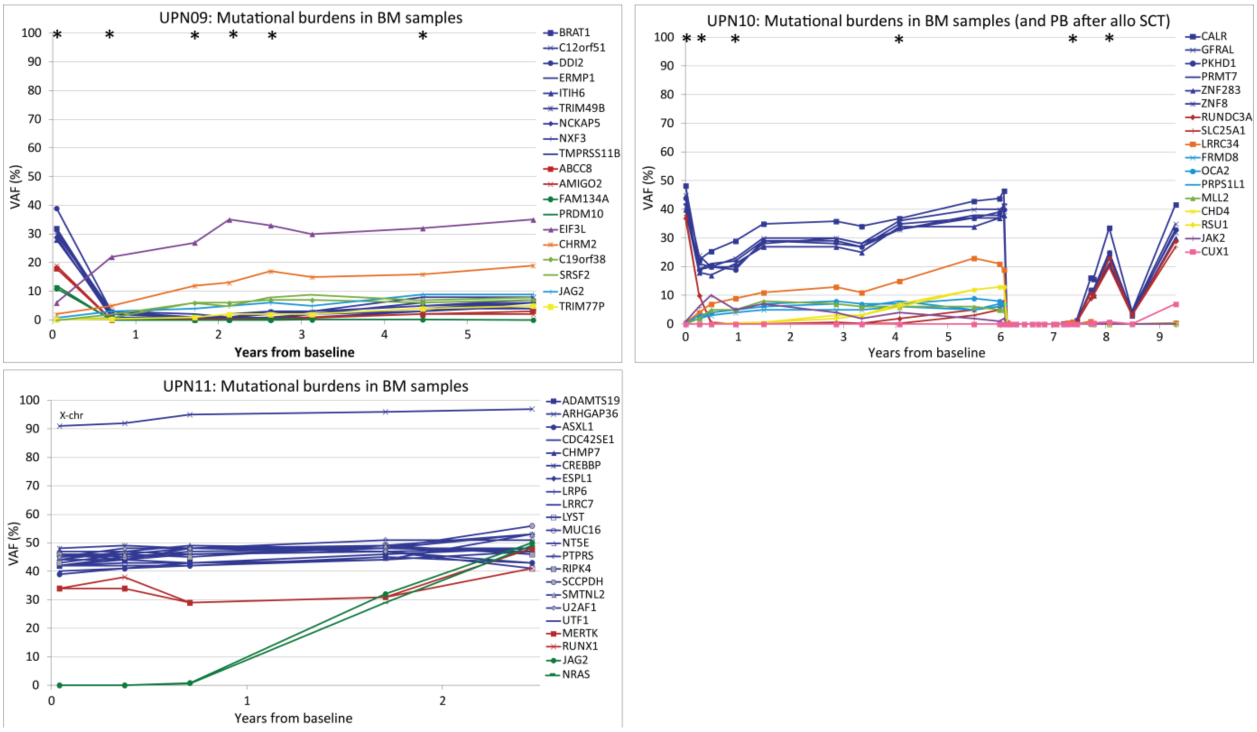

Supplementary Figure 4. Mutational burdens in bone marrow (BM) samples. Graphs showing the variant allele frequencies (VAFs) measured using amplicon-based targeted deep sequencing (not corrected for local ploidy). For patient UPN09, 10 and 11, the patterns of all mutations are plotted in time and mutations are color-coded to match the (sub)clones as depicted in figures 2 and 3. Indicated is also whether a variant locates to the X-chromosome in case of a male patient. *indicates amplified DNA sample used for targeted deep sequencing

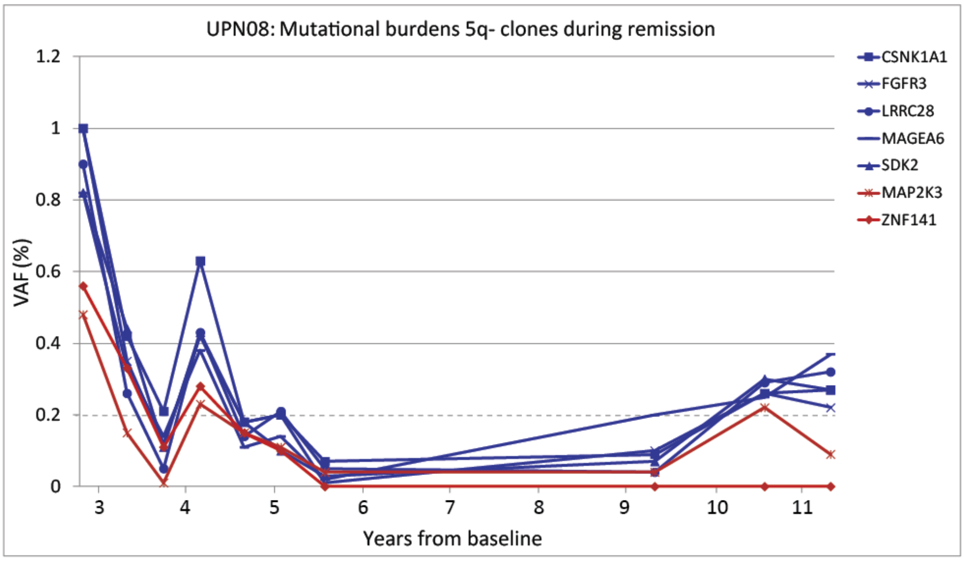

Supplementary Figure 5. Mutations still detectable in remission time points of patient UPN08. Mutations can still be detected at low variant allele frequency (VAF:~0.2%) under lenalidomide treatment, which indicates the presence of remaining MDS cells (~0.4%).

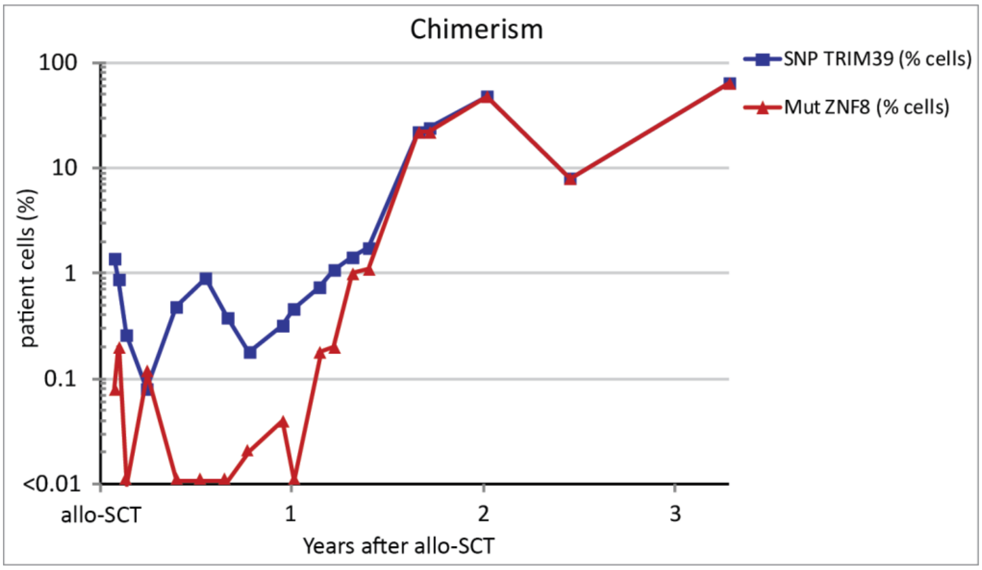

Supplementary Figure 6. Chimerism analysis in peripheral blood samples of UPN10 after allogeneic stem cell transplantation (allo-SCT). The percentage of blood cells with a patient-specific single nucleotide polymorphism (SNP TRIM39) is depicted, as well as the percentage of blood cells with a patient-specific mutation (mut ZNF8). Patient cells could still be detected after allo-SCT (<1%). Approximately one year after SCT, the mutated cells started to increase in frequency, causing a relapse of MDS.

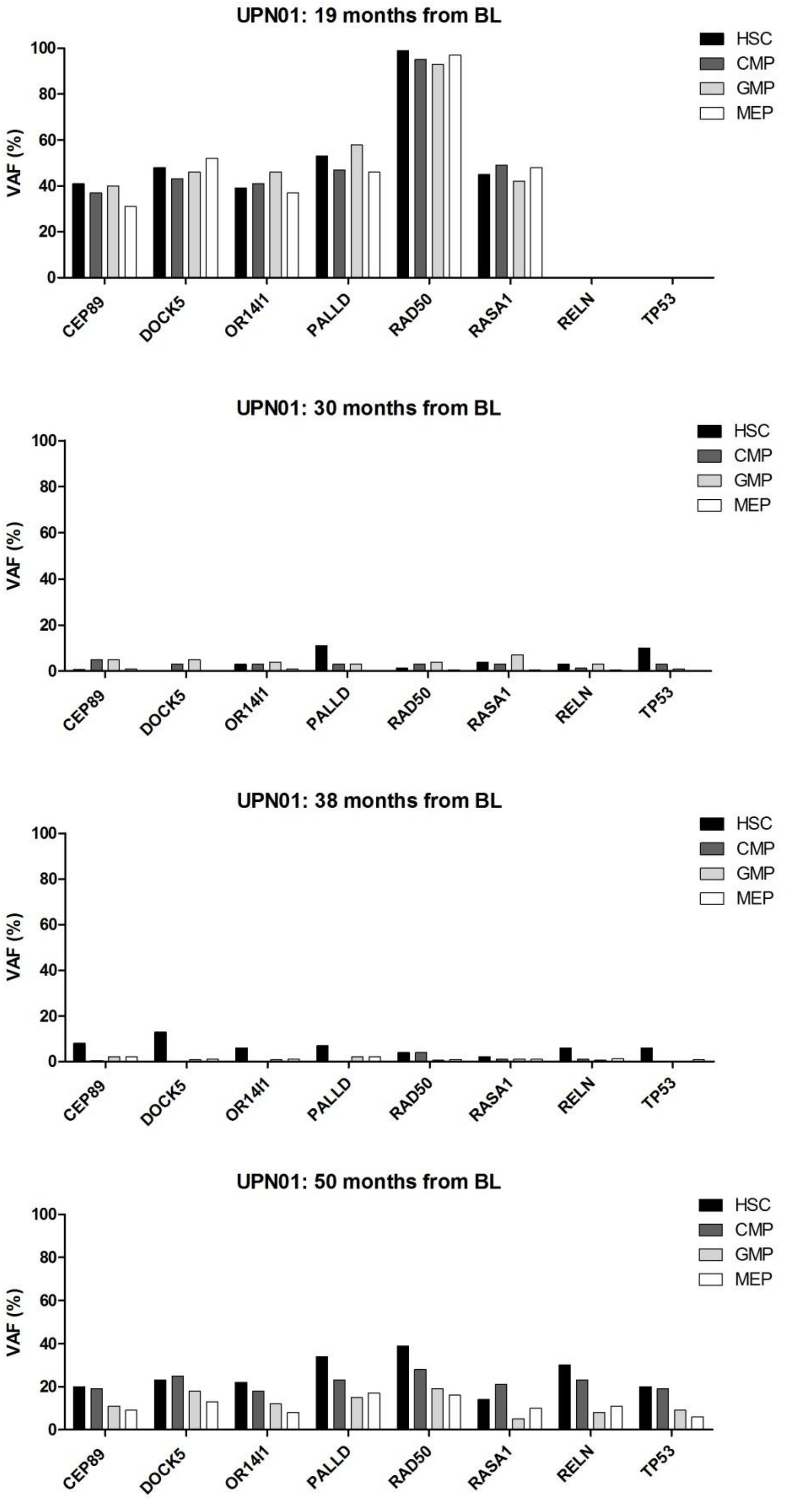

Supplementary Figure 7. Comparison of variant allele frequencies (VAFs) in myeloid progenitor fractions of UPN01. Hematopoietic stem cells (HSCs), common myeloid progenitors (CMPs), granulocyte-macrophage progenitors (GMPs) and megakaryocyte-erythroid progenitors (MEPs) were sorted from bone marrow samples. The VAFs in all different progenitor fractions are compared. VAFs are not corrected for local ploidy. BL indicates baseline.

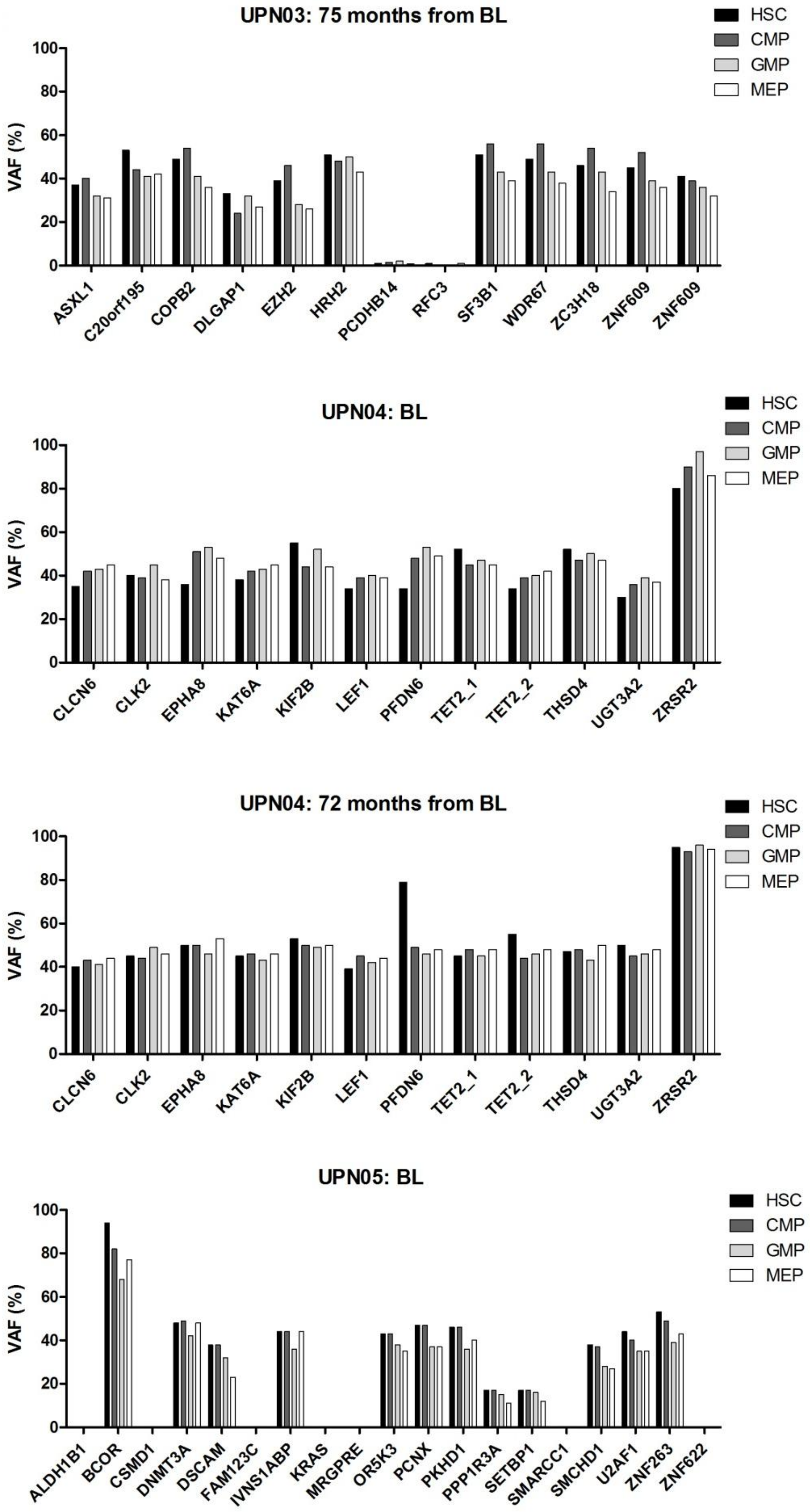

Supplementary Figure 8. Comparison of variant allele frequencies (VAFs) in myeloid progenitor fractions of UPN03, 04 and 05. Hematopoietic stem cells (HSCs), common myeloid progenitors (CMPs), granulocyte-macrophage progenitors (GMPs) and megakaryocyte-erythroid progenitors (MEPs) were sorted from bone marrow samples. The VAFs in all different progenitor fractions are compared. VAFs are not corrected for local ploidy. BL indicates baseline

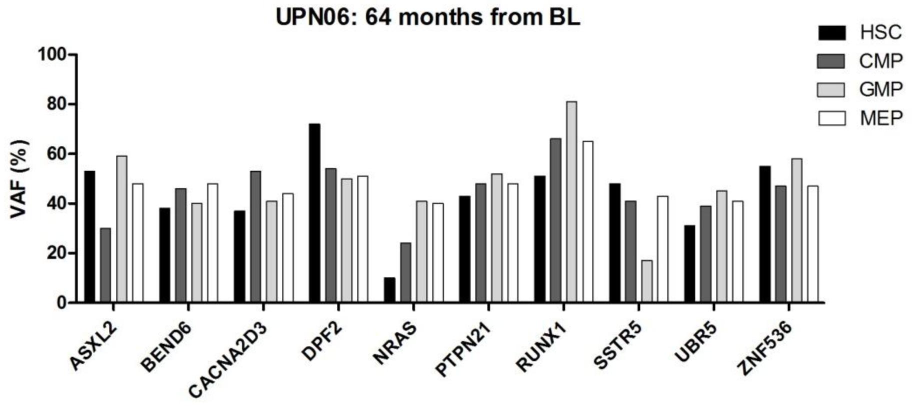

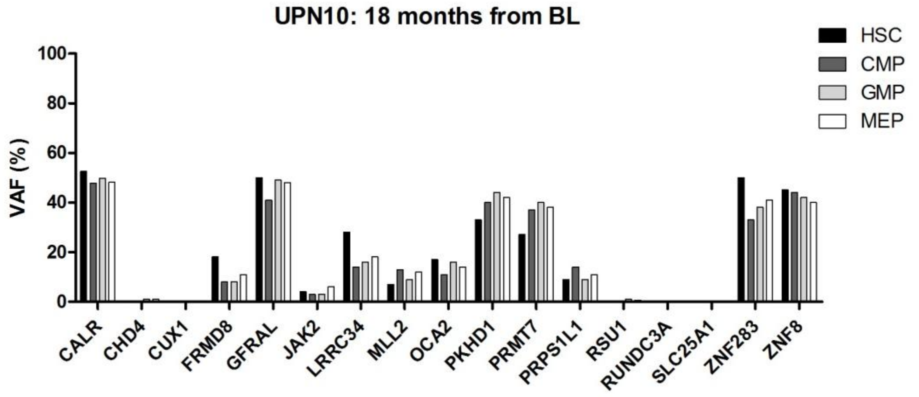

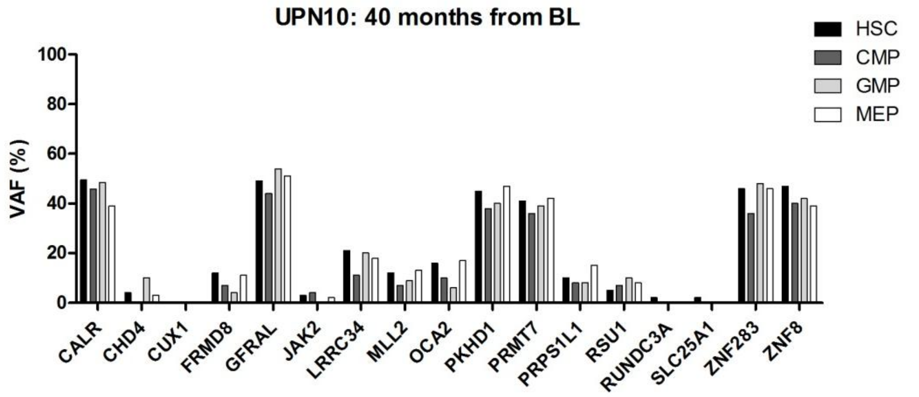

Supplementary Figure 9. Comparison of variant allele frequencies (VAFs) in myeloid progenitor fractions of UPN06 and 10. Hematopoietic stem cells (HSCs), common myeloid progenitors (CMPs), granulocyte-macrophage progenitors (GMPs) and megakaryocyte-erythroid progenitors (MEPs) were sorted from bone marrow samples. The VAFs in all different progenitor fractions are compared. VAFs are not corrected for local ploidy. BL indicates baseline

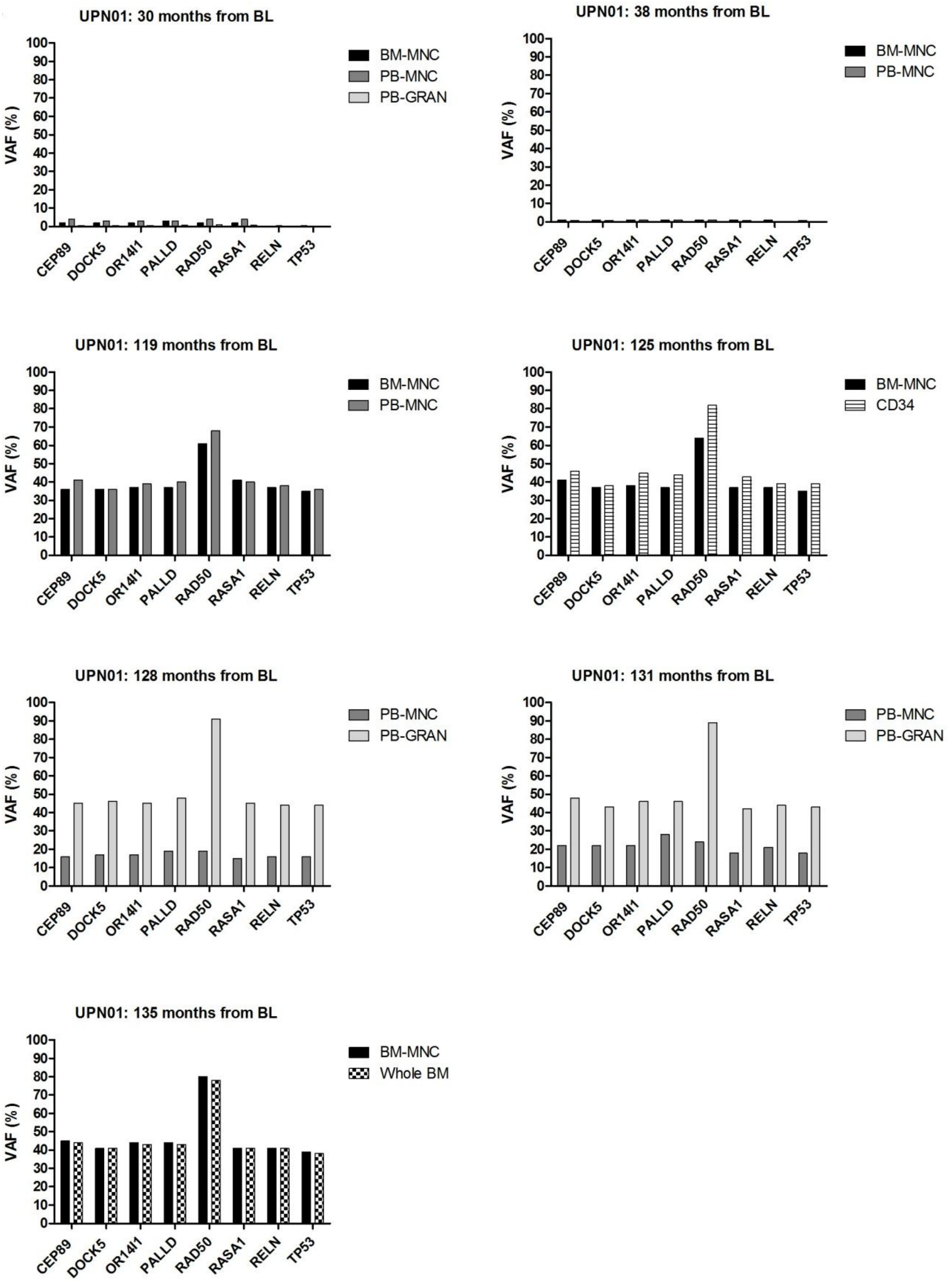

Supplementary Figure 10. Comparison of variant allele frequencies (VAFs) in different peripheral blood (PB) and bone marrow (BM) fractions of UPN01. For some time points, multiple PB or BM fractions were analyzed. The comparison of different fractions per time point is depicted. VAFs are not corrected for local ploidy. BL indicates baseline.

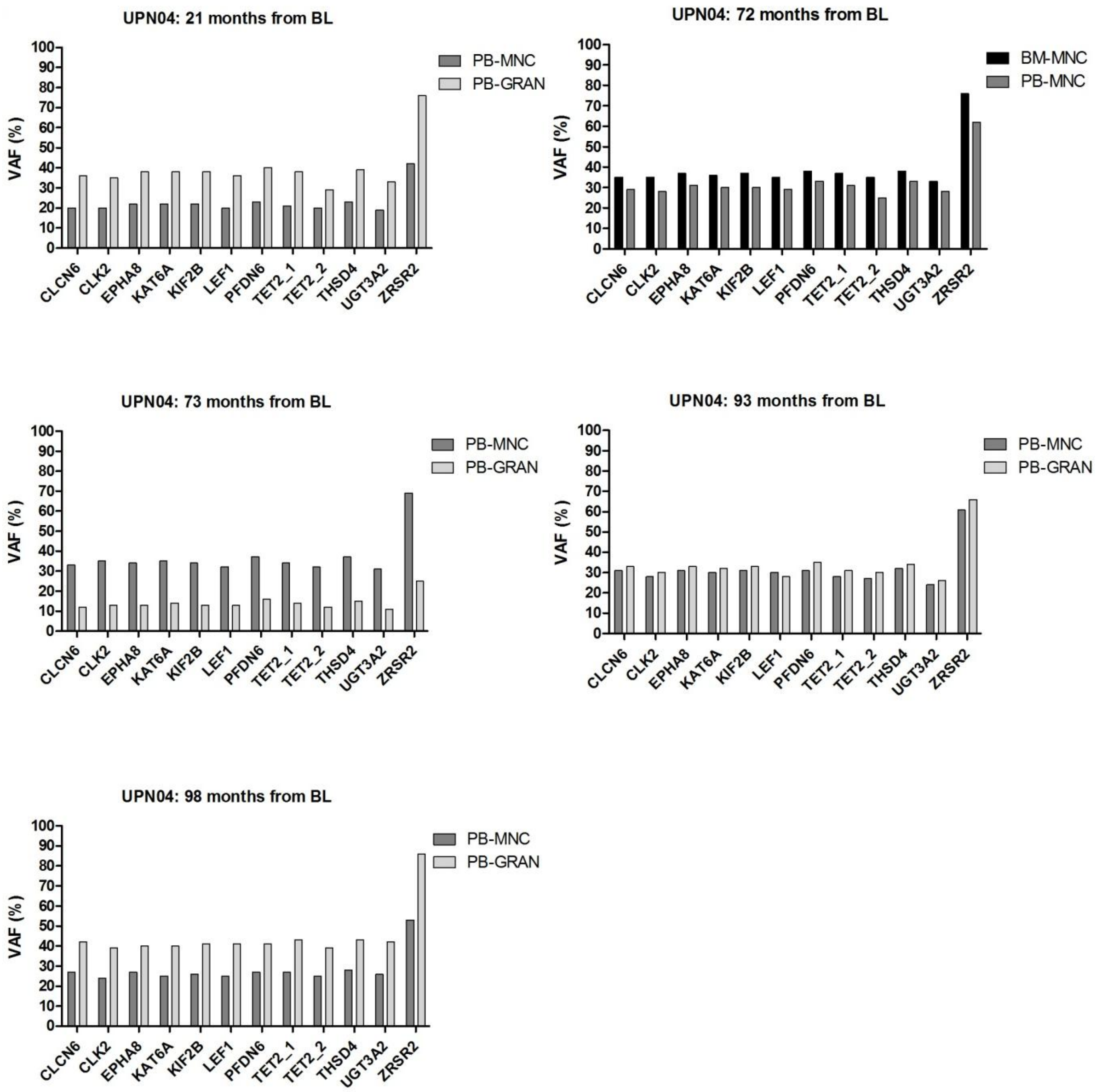

Supplementary Figure 11. Comparison of variant allele frequencies (VAFs) in different peripheral blood (PB) and bone marrow (BM) fractions of UPN04. For some time points, multiple PB or BM fractions were analyzed. The comparison of different fractions per time point is depicted. VAFs are not corrected for local ploidy. BL indicates baseline.

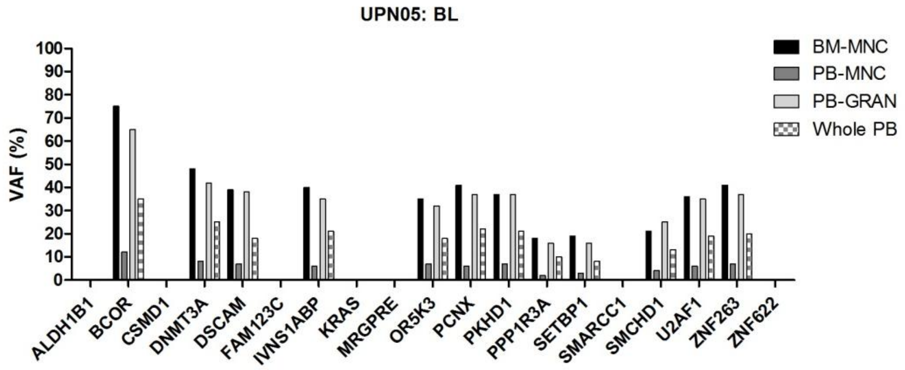

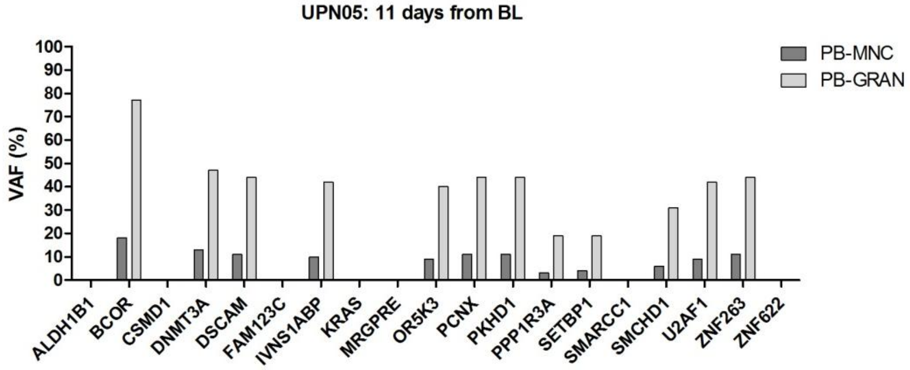

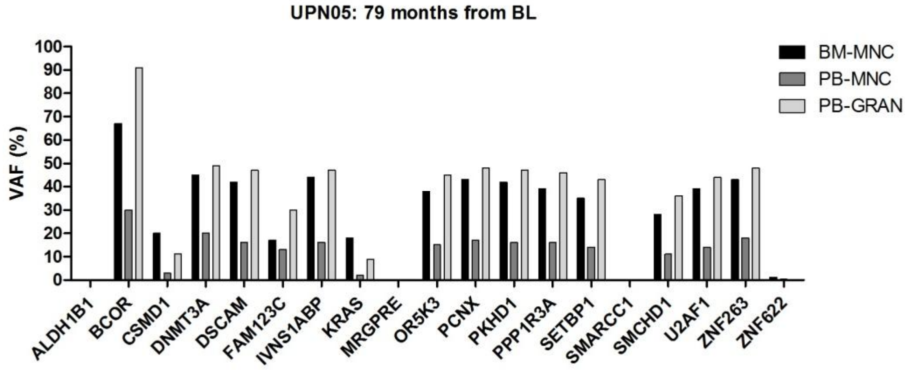

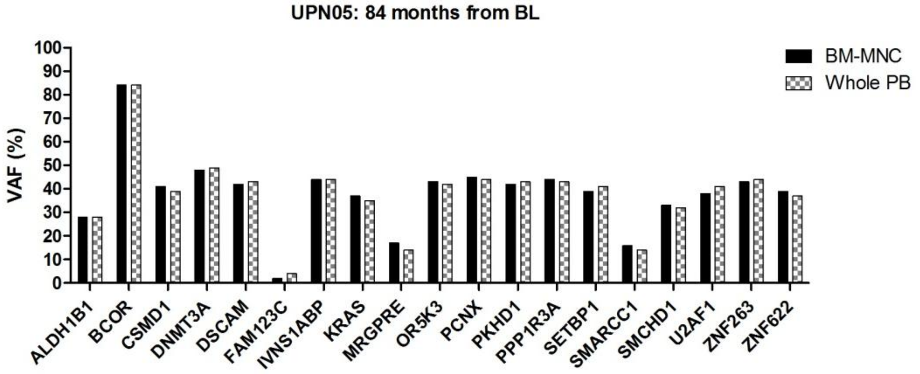

Supplementary Figure 12. Comparison of variant allele frequencies (VAFs) in different peripheral blood (PB) and bone marrow (BM) fractions of UPN05. For some time points, multiple PB or BM fractions were analyzed. The comparison of different fractions per time point is depicted. VAFs are not corrected for local ploidy. BL indicates baseline.

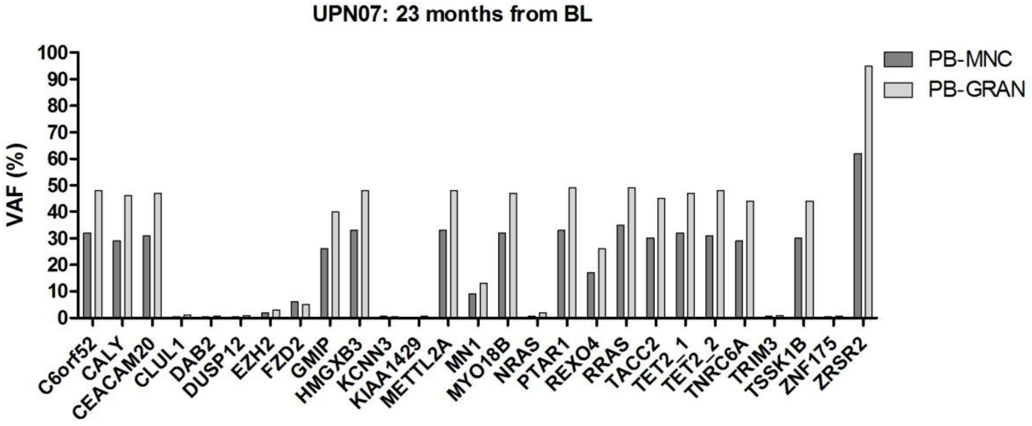

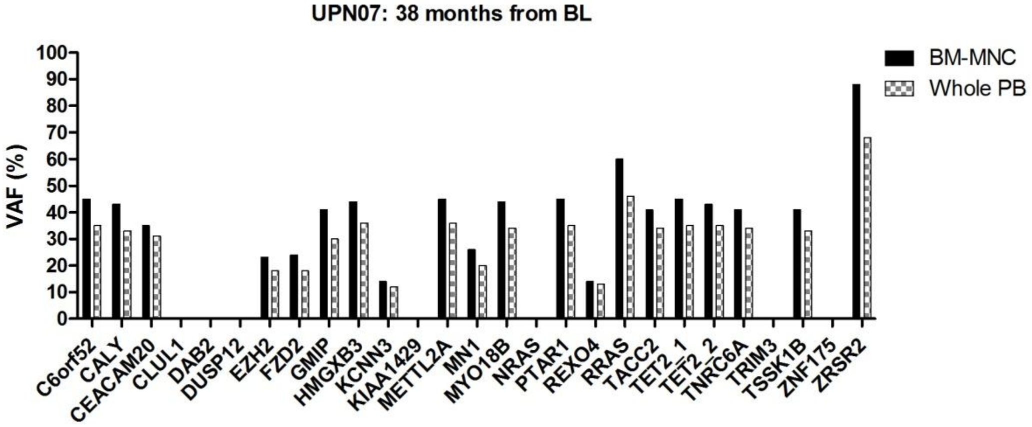

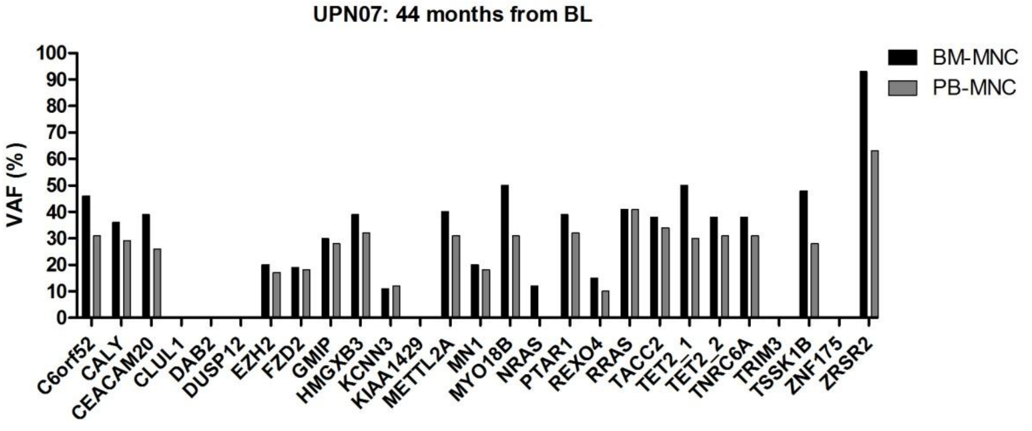

Supplementary Figure 13. Comparison of variant allele frequencies (VAFs) in different peripheral blood (PB) and bone marrow (BM) fractions of UPN07. For some time points, multiple PB or BM fractions were analyzed. The comparison of different fractions per time point is depicted. VAFs are not corrected for local ploidy. BL indicates baseline.

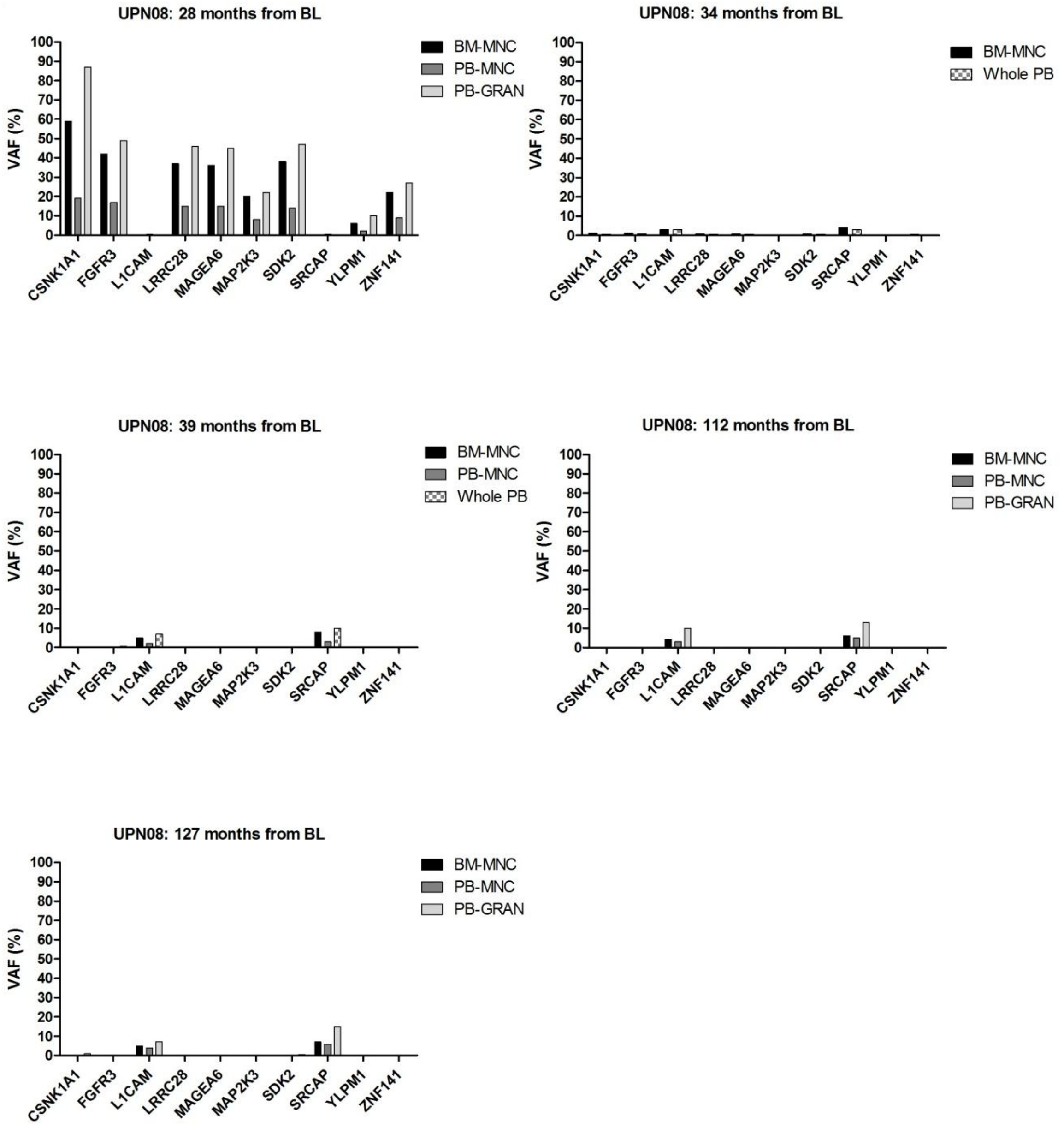

Supplementary Figure 14. Comparison of variant allele frequencies (VAFs) in different peripheral blood (PB) and bone marrow (BM) fractions of UPN08. For some time points, multiple PB or BM fractions were analyzed. The comparison of different fractions per time point is depicted. VAFs are not corrected for local ploidy. BL indicates baseline.

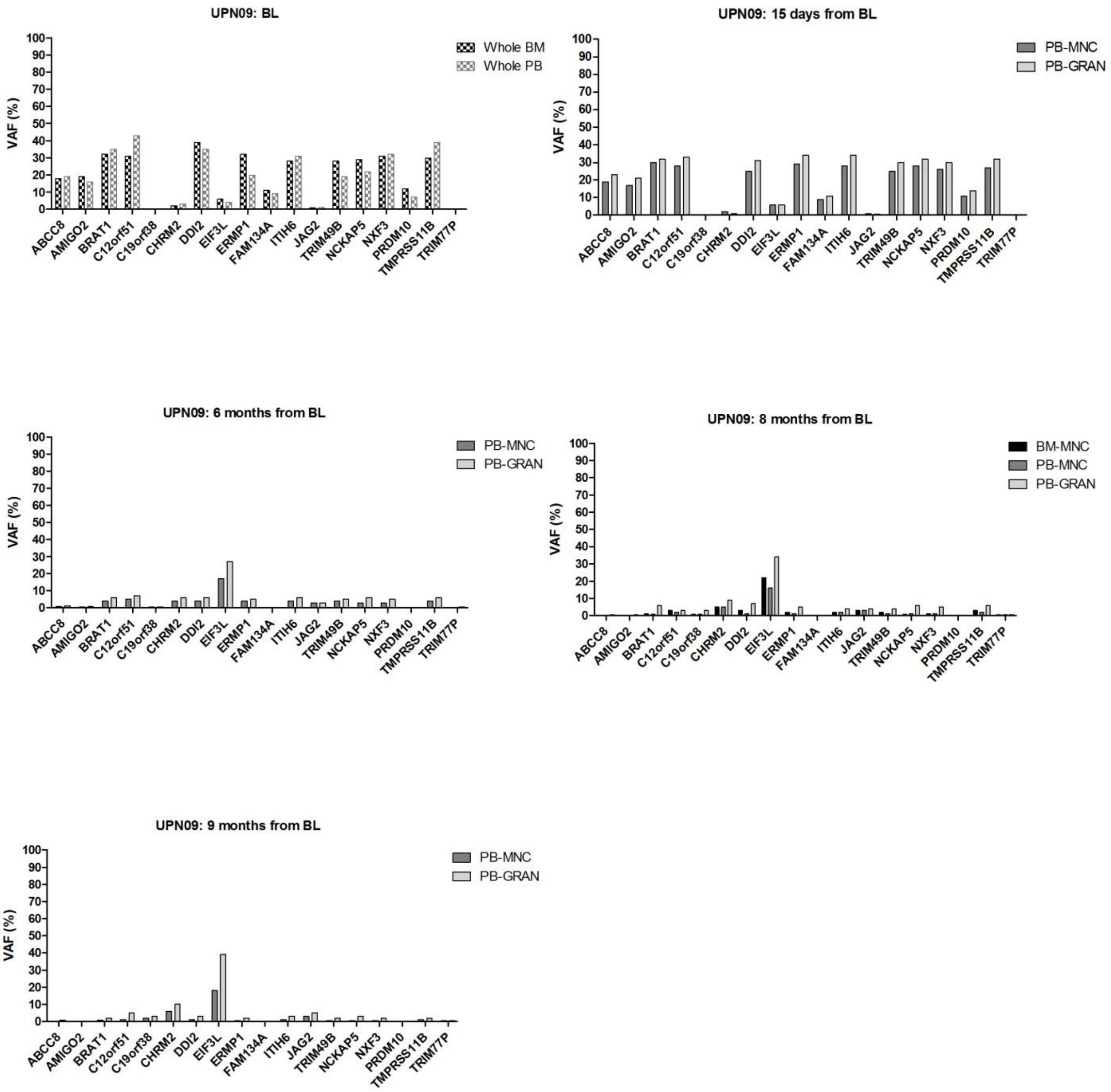

Supplementary Figure 15. Comparison of variant allele frequencies (VAFs) in different peripheral blood (PB) and bone marrow (BM) fractions of UPN09. For some time points, multiple PB or BM fractions were analyzed. The comparison of different fractions per time point is depicted. VAFs are not corrected for local ploidy. BL indicates baseline.

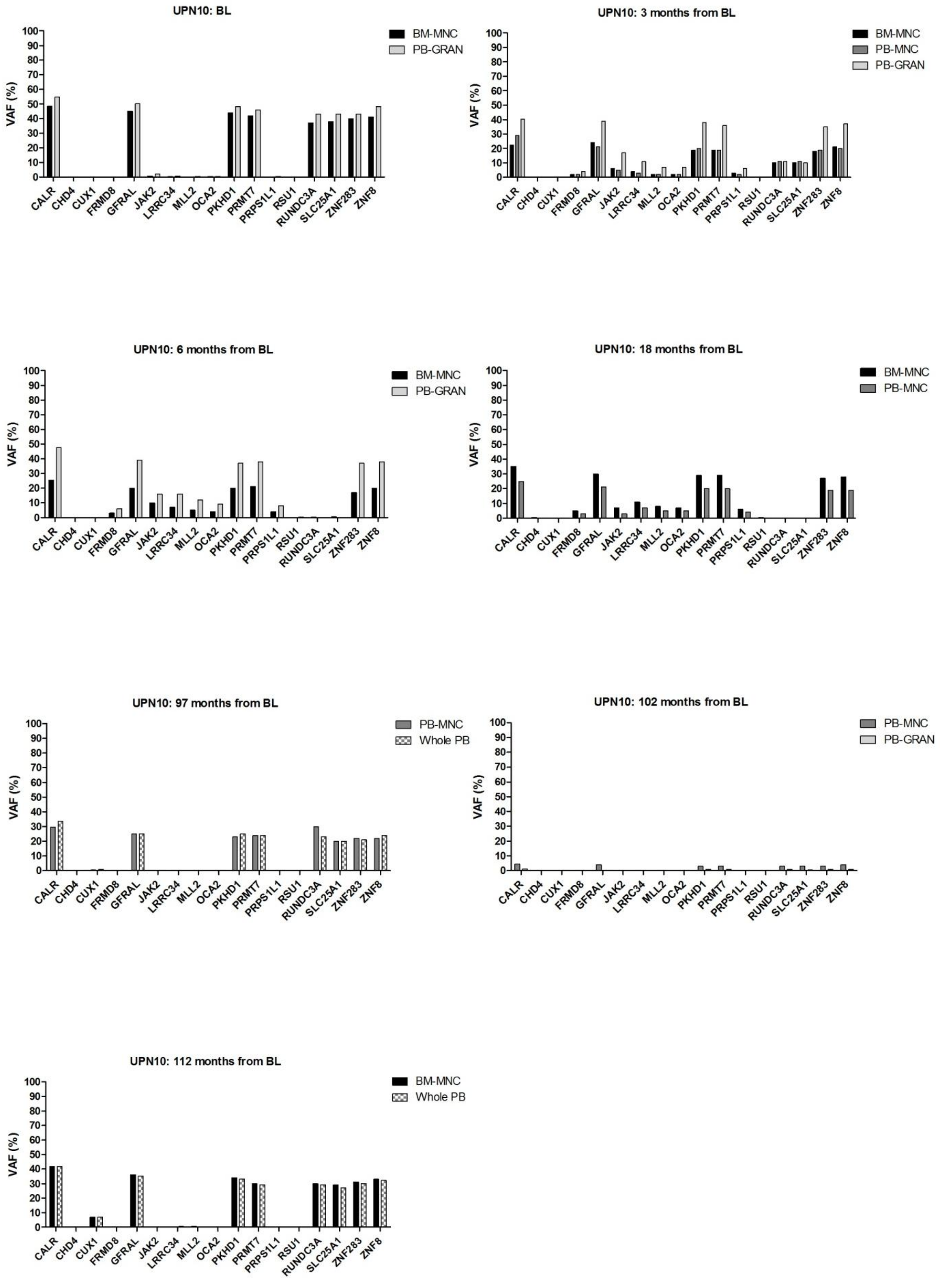

Supplementary Figure 16. Comparison of variant allele frequencies (VAFs) in different peripheral blood (PB) and bone marrow (BM) fractions of UPN10. For some time points, multiple PB or BM fractions were analyzed. The comparison of different fractions per time point is depicted. VAFs are not corrected for local ploidy. BL indicates baseline.

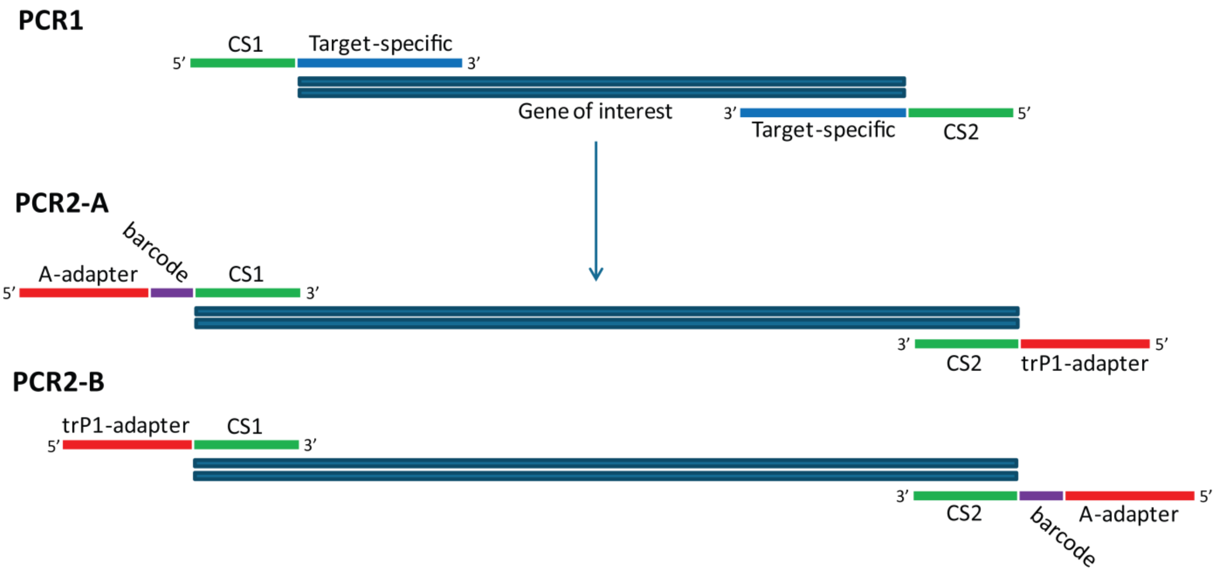

Supplementary Figure 17. Primer strategy targeted deep sequencing. In PCR1 target fragments are amplified and tagged with common sequence (CS) tags. In PCR2, primers containing a CS-tag, a barcode and an adapter are used to label the PCR fragments with a sample-specific Ion Xpress barcode. The second PCR is performed twice, once with the A adapter attached to the forward primer and the truncated P1 (trP1) adapter to the reverse primer (PCR2-A) and vice versa (PCR2-B), making bidirectional sequencing possible.
